# Random Beam-Based Non-Orthogonal Multiple Access for Low Latency K-User MISO Broadcast Channels

**DOI:** 10.3390/s21134373

**Published:** 2021-06-26

**Authors:** Jung Hoon Lee, Yunjoo Kim, Jong Yeol Ryu

**Affiliations:** 1 Department of Electronics Engineering and Applied Communications Research Center, Hankuk University of Foreign Studies, Yongin 17035, Korea; tantheta@hufs.ac.kr; 2 Telecommunications & Media Research Laboratory, Electronics and Telecommunications Research Institute, Daejeon 34129, Korea; yunjoo@etri.re.kr; 3 Department of Information and Communications Engineering, Gyeongsang National University, Tongyeong 53064, Korea

**Keywords:** random beamforming, non-orthogonal multiple access (NOMA), multiple-input single-output (MISO), broadcast channels (BC)

## Abstract

In this paper, we propose random beam-based non-orthogonal multiple access (NOMA) for low latency multiple-input single-output (MISO) broadcast channels, where there is a target signal-to-interference-plus-noise power ratio (SINR) for each user. In our system model, there is a multi-antenna transmitter with its own single antenna users, and the transmitter selects and serves some of them. For low latency, the transmitter exploits random beams, which can reduce the feedback overhead for the channel acquisition, and each beam can support more than a single user with NOMA. In our proposed random beam-based NOMA, each user feeds a selected beam index, the corresponding SINR, and the channel gain, so it feeds one more scalar value compared to the conventional random beamforming. By allocating the same powers across the beams, the transmitter independently selects NOMA users for each beam, so it can also reduce the computational complexity. We optimize our proposed scheme finding the optimal user grouping and the optimal power allocation. The numerical results show that our proposed scheme outperforms the conventional random beamforming by supporting more users for each beam.

## 1. Introduction

In recent years, various forms of wireless applications have emerged as the performance of wireless communication systems significantly improved. This includes a variety of wireless applications such as real-time remote control, inter-vehicle communication, autonomous driving, and augmented reality, most of which require high reliability and low latency characteristics. In the cases of wireless factory system control, inter-vehicle communication, and autonomous driving, it is very important to satisfy high reliability and low-latency characteristics because transmission errors or delays can cause great damage or risk. To support these wireless applications, the upcoming beyond-5G communication system defines a variety of target performances, including end-to-end communication delay of 1 ms, 10 Gbps transmission rate, and a 90% reduction in energy usage [1]. Furthermore, a significant increase in the number of wireless devices brings challenges to the environment, where many devices directly communicate with each other, which is sensitive to latency. Thus, it becomes very important to investigate wireless transmission technologies that serve large numbers of devices with low latency.

According to the CISCO report [2], the number of device-to-device (D2D) connections is expected to reach 14.7 billion by 2023. As the number of devices participating in wireless networks rapidly increases, various technologies are being studied to support the communication of a large number of devices, which triggers various problems such as latency and increased signaling complexity. The latency of wireless communication systems can be divided into (1) end-to-end latency (2) user plane latency (3) control plane latency [3,4]. End-to-end latency is comprised of wireless propagation delay, processing delay, queuing delay, retransmission delay, and computational delay. Furthermore, user plane latency is defined as the time spent transmitting a single message from the transmitter’s application layer to the receiver’s application layer. Control plane latency is defined as the amount of time the terminal takes to activate. Meanwhile, reliability is usually defined as the successful transmission probability of a certain size of a message in a given time [5].

One way to support a large number of devices is non-orthogonal multiple access (NOMA) [6,7,8], which allows multiple users to share the same radio resources unlike the conventional orthogonal multiple access (OMA) that exclusively uses the radio resources. The NOMA can be classified into two categories: power-domain NOMA and code-domain NOMA. In the case of downlink power-domain NOMA, the transmitter uses different powers to serve multiple users. In power-domain NOMA, the transmitter simply transmits the superposed one of the users’ signals. Then, a user with a better channel can decode the other users’ signals, so it can subtract them from the received signal, i.e., successive interference cancelation (SIC). In this case, the transmitter allocates smaller power to the user with better channel.

The NOMA is widely studied in many scenarios. The authors of [9] proposed intelligent reflecting surface (IRS)-assisted NOMA to support cell edge users in cellular systems. The authors of [10,11] exploit machine learning techniques to optimize NOMA. Furthermore, the authors of [12] considered uplink cellular communication scenarios and analysed the ergodic sum rate gain of NOMA compared to orthogonal multiple access (OMA). The authors of [13] proposed the uplink network NOMA scheme for the uplink coordinated multi-point transmission (CoMP), where a CoMP user and multiple NOMA users are served simultaneously. Meanwhile, the authors of [14] proposed the resource allocation scheme for NOMA to guarantee the quality of service (QoS) in multibeam satellite industrial Internet of things, and the authors of [15] adopted NOMA for multiple-input multiple-output (MIMO) multi-user visible light communication systems.

In this paper, we propose random beam-based non-orthogonal multiple access for low latency multiple-input single-output (MISO) broadcast channels, where there is a target signal-to-interference-plus-noise power ratio (SINR) for each user. In our system model, there is a multi-antenna transmitter with its own single antenna users, and the transmitter selects and serves some of them. For low latency, the transmitter exploits random beams [16], which can reduce the feedback overhead for the channel acquisition, and each beam can support more than a single user with NOMA. The basic idea of random beam-based NOMA is presented in [17], but in [17], we mainly consider a simple case in which each beam can support at most two users with equal powers, and the exact power allocation for each beam is not revealed. Our contributions can be summarized as follows:

- We propose random beam-based NOMA generalizing the basic idea of [17], where each beam can support multiple users with the optimal power allocation. We identify the feedback information for each user to implement random beam-based NOMA; each user should feed (1) a selected beam index, (2) the corresponding SINR, and (3) the channel gain, while the conventional random beamforming requires (1) a selected beam index and (2) the corresponding SINR feedback for each user.
- We formulate a joint user selection and power optimization problem for random beam-based NOMA and show that equal power allocation across the beams can reduce the computational complexity and reduces the feedback overhead.
- With the equal power allocation across the beams, we show that our optimization problem can be divided into sub-optimization problems at all beams. We solve each sub-optimization problem and find the optimal user selection and power allocation.
- In the simulation part, we evaluate our random beam-based NOMA and show that our proposed scheme well exploits multiuser diversity provided by the multiple users and increases the performance of the conventional random beamforming.

The remainder of this paper is organized as follows. In Section 2, we explain our system model and summarize the conventional random beamforming with a QoS constraint. In Section 3, we propose random beam-based NOMA, and in Section 4, we optimize our proposed scheme. In Section 5, we evaluate our proposed scheme, and in Section 6, we conclude our paper.

## 2. System Model

**Figure 1:**
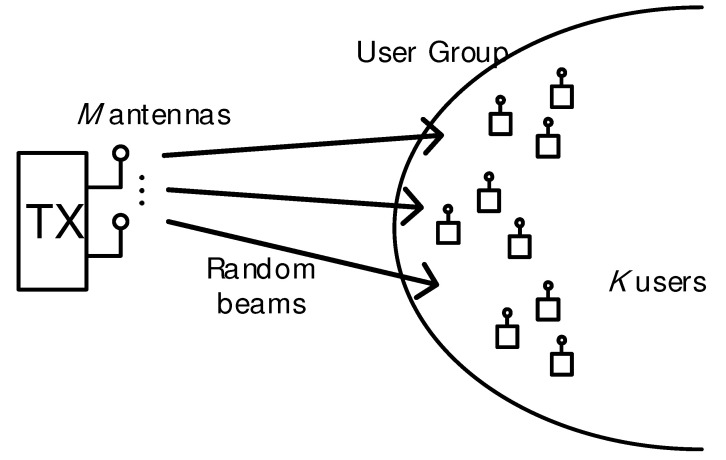
System model.

Figure 1 illustrates our system model. There is a single transmitter equipped with *M* antennas with its own *K* single-antenna users, among which the transmitter selects and serves some of them. Let G⊂{1,…,K} be a user group selected at the transmitter, where [*K*] is the set of integers less than or equal to *K*, i.e., [*n*] ≜ {1,…, *K*}. %. Then, the received signal at the arbitrary selected user k∈G becomes

(1) $$\begin{array}{c} y_{k}=h_{k}^{†}x+n_{k}, \end{array}$$

where $h_{k}\in C^{M\times 1}$ is a channel between the transmitter and the user *k*, whose elements are independent and identically distributed (i.i.d.) circularly symmetric complex Gaussian random variables with zero mean and unit variance, i.e., $h_{k}∼\mathrm{CN}(0,I_{M})$. Furthermore, $x\in C^{M\times 1}$ is the transmitted signal at the transmitter, and $n_{k}$ is an additive white complex Gaussian noise at the user *k* with zero mean and unit variance, i.e., $n_{k}∼\mathrm{CN}\left(0,1\right)$. Meanwhile, we assume that the transmitter exploits linear beamforming vectors to serve the selected users, so the transmitted signal is constructed by

(2) $$\begin{array}{c} x=\sum_{i\in G}v_{i}x_{i}, \end{array}$$

where $v_{i}\in C^{M\times 1}$ is a beamforming vector for the user *i* such that $∥v_{i}{∥}^{2}=1$, and $x_{i}$ is a data symbol for the user *i* such that $E|x_{i}{|}^{2}=P_{i}$ with $P_{i}$ the power allocation for user *i*. Denoting by *P* the transmitter’s total power budget, it should be satisfied that $tr(xx^{†})=P$.

### Conventional Random Beamforming with a QoS Constraint

To enjoy the multiplexing gain, the transmitter should exploit the channel state information (CSI). The CSI acquisition at the transmitter is not easy in practice, however, and the imperfect CSI causes the severe performance degradation. One way to circumvent this difficulty is to use random beams with user diversity in opportunistic manners.

The authors of [16] proposed random beamforming and showed that the random beamforming can achieve the optimal multiplexing gain when the number of users increases with the transmit power. The procedure of the conventional random beamforming can be summarized as follows:

- The transmitter broadcasts *M* random beams to the users.
- Each user chooses the best beam and feeds the beam index and one scalar value, which represents the performance of the selected beam, back to the transmitter. In this case, it is assumed that perfect CSI is available at users.
- From the collected feedback information, the transmitter selects multiple users.
- The transmitter serves the selected users with random beams. In this case, the transmitter selects a *single* user for each beam and allocates equal power to the selected users at all beams.

For the conventional random beamforming, the transmitter exploits *M* orthogonal random beams $v_{1},\cdots ,v_{M}\in C^{M}$ such that $v_{i}⊥v_{j}$ whenever i≠j. In this case, we assume that each random beam is a unit vector, i.e., $∥v_{m}{∥}^{2}=1$ for all m∈[M].

After the transmitter’s random beam broadcasting, each user finds the closest beam to its own channel; the user *k* returns I(k), where I(k) is a selected beam indicator for the user *k* given by

(3) $$\begin{array}{c} I\left(k\right)=\underset{i\in \{1,\ldots ,K\}}{\arg\max}\;\;{\left|h_{k}^{†}v_{i}\right|}^{2}. \end{array}$$

When the user *k* is served by the *m*th random beam, i.e., $v_{m}$, and the transmitter allocates equal power to the selected users, the signal-to-interference-plus-noise power ratio (SINR) of the user *k* becomes

(4) $$\begin{array}{c} {\mathrm{SINR}}_{k}=\frac{\frac{P}{M}{\left|h_{k}^{†}v_{m}\right|}^{2}}{\sum_{i\in [M]\backslash \{m\}}\frac{P}{M}{\left|h_{k}^{†}v_{i}\right|}^{2}+1}. \end{array}$$

In this case, the user *k*’s signal-to-noise power ratio (SNR) becomes

(5) $$\begin{array}{c} \Gamma_{k}≜{\left|h_{k}^{†}v_{I(k)}\right|}^{2}, \end{array}$$

while the user *k*’s interference-to-noise power ratio (INR) becomes

(6) $$\begin{array}{c} {\mathrm{INR}}_{k}≜\sum_{i\in [M]\backslash \{m\}}\frac{P}{M}{\left|h_{k}^{†}v_{i}\right|}^{2}. \end{array}$$

Thus, the achievable rate when the user *k* is served by the *m*th beam is given by

(7) $$\begin{array}{c} \log_{2}\left(1+{\mathrm{SINR}}_{k}\right), \end{array}$$

and the sum achievable rate from the selected users becomes

(8) $$\begin{array}{c} \sum_{i\in G}\log_{2}\left(1+{\mathrm{SINR}}_{i}\right). \end{array}$$

To maximize (8), the user *k* feeds the selected beam and the corresponding SINR as follows:

(9) $$\begin{array}{c} \left\{I\left(k\right),\;\;{\mathrm{SINR}}_{k}\right\} \end{array}$$

After collecting the feedback values from the users, i.e., ${\left\{I\left(k\right),{\mathrm{SINR}}_{k}\right\}}_{k=1}^{K}$, the transmitter selects the best user for each beam, which can achieve the highest SINR with the beam.

Let $s_{m}$ be the selected user index from the beam *m*. Then, $s_{m}$ can be obtained as follows

(10) $$\begin{array}{c} s_{m}=\underset{k}{\arg\max}\;\left\{{\mathrm{SINR}}_{k}\;|\;I\left(k\right)=m\right\}, \end{array}$$

so the SINR at the *m*th beam becomes

(11) $$\begin{array}{c} {\mathrm{SINR}}_{s_{m}}=\frac{P_{s_{m}}{\left|h_{s_{m}}^{†}v_{m}\right|}^{2}}{\sum_{i\in [M]\backslash \{m\}}P_{s_{i}}{\left|h_{s_{m}}^{†}v_{i}\right|}^{2}+1}. \end{array}$$

In this paper, we consider a quality of service (QoS) constraint, where each user’s SINR should exceed a target SINR denoted by γ. Then, the sum achievable rate at the transmitter becomes

(12) $$\begin{array}{c} R_{\mathrm{sum}}^{\mathrm{OMA}}=\sum_{m=1}^{M}\left[1\left({\mathrm{SINR}}_{s_{m}}\geq \gamma \right)\cdot \log_{2}\left(1+\gamma \right)\right], \end{array}$$

where 1(·) is the indicator function that returns one when the event happens and zero otherwise.

## 3. Non-Orthogonal Multiple Access with Random Beamforming

In this paper, we implement non-orthogonal multiple access with random beamforming, where each beam can serve more than a single user. When the transmitter serves multiple users with the same beam, the transmitter simply superposes the transmitted signal, and the multiple users adopt the successive interference cancelation (SIC).

As each random beam can serve multiple users, we denote by $G_{m}\in \left[K\right]$ the set of users served with the *m*th random beam, i.e., $v_{m}$, and assume that the sub-user groups $G_{1},\ldots ,G_{M}$ become pairwise disjoint sets such that

(13) $$\begin{array}{c} G_{i}\cap G_{j}=\phi \;\;\mathrm{whenever}\;\;i\neq j. \end{array}$$

Then, the transmitted signal x given in (2) changes to

(14) $$\begin{array}{c} x=\sum_{m=1}^{M}\sum_{s\in G_{m}}v_{m}x_{s}, \end{array}$$

where the power constraint becomes

(15) $$\begin{array}{c} \sum_{m=1}^{M}\sum_{s\in G_{m}}E{\left|x_{s}\right|}^{2}=P. \end{array}$$

In this case, the received signal at the user *s* served from the *m*th beam, i.e., $s\in G_{m}$, becomes as follows:

(16) $$\begin{array}{cc} y_{s} & =h_{s}^{†}v_{m}x_{s}+h_{s}^{†}v_{m}\left(\sum_{i\in G_{m}\backslash \left\{s\right\}}x_{i}\right)+h_{s}^{†}\left(\sum_{i\in [M]\backslash \{m\}}\sum_{j\in G_{i}}v_{i}x_{j}\right)+n_{s}, \end{array}$$

where $h_{s}^{†}v_{m}x_{s}$ is the user *s*’s desired signal, and $h_{s}^{†}v_{m}\left(\sum_{i\in G_{m}\backslash \left\{k\right\}}x_{i}\right)$ is the interference within the same beam, i.e., the intra-beam interference. Furthermore, the term $h_{s}^{†}\left(\sum_{i\in [M]\backslash \{m\}}\sum_{j\in G_{i}}v_{i}x_{j}\right)$ is the interference from other beams, i.e., the inter-beam interference.

With the NOMA, the interference within the same beam is managed with the SIC of the users. To denote the decoding order at the user group $G_{m}$, we define a sequence that is a permutation of a sequence of all user indexes belonging to $G_{m}$ as follows:

(17) $$\begin{array}{c} \pi_{m}≜\left[\pi_{m}^{\left(1\right)},\ldots ,\pi_{m}^{\left(|G_{m}|\right)}\right], \end{array}$$

where |·| is the cardinality of a set. Meanwhile, without loss of generality, we assume that

(18) $$\begin{array}{c} {\mathrm{SINR}}_{\pi_{m}^{\left(i\right)}}\geq {\mathrm{SINR}}_{\pi_{m}^{\left(j\right)}}\;\;\mathrm{whenever}\;\;i\leq j. \end{array}$$

Then, for any (i,j) such that $1\leq i\leq j\leq |G_{m}|$, the user $\pi_{m}^{\left(i\right)}$ can decode the user $\pi_{m}^{\left(j\right)}$’s signal, so it can subtract it from the received signal.

Now, we consider the user $\pi_{m}^{\left(l\right)}$ in the user group $G_{m}$. From (16), we can rewrite the user $\pi_{m}^{\left(l\right)}$’s received signal as follows:

(19) $$\begin{array}{cc} y_{\pi_{m}^{\left(l\right)}}\; & =h_{\pi_{m}^{\left(l\right)}}^{†}v_{m}x_{\pi_{m}^{\left(l\right)}}+h_{\pi_{m}^{\left(l\right)}}^{†}v_{m}\left(\sum_{i=1}^{\pi_{m}^{\left(l-1\right)}}x_{i}\right)+h_{\pi_{m}^{\left(l\right)}}^{†}v_{m}\left(\sum_{i=\pi_{m}^{\left(l+1\right)}}^{\pi_{m}^{\left(|G_{m}|\right)}}x_{i}\right)\\ \; & \;+h_{\pi_{m}^{\left(l\right)}}^{†}\left(\sum_{i\in [M]\backslash \{m\}}\sum_{j\in G_{i}}v_{i}x_{j}\right)+n_{\pi_{m}^{\left(l\right)}}. \end{array}$$

Then, the user $\pi_{m}^{\left(l\right)}$ can decode the signal of the users $\pi_{m}^{\left(l+1\right)},\ldots ,\pi_{m}^{\left(|G_{m}|\right)}$ from the received signal, so after SIC, the received signal (19) becomes

(20) $$\begin{array}{cc} y_{\pi_{m}^{\left(l\right)}}\; & =h_{\pi_{m}^{\left(l\right)}}^{†}v_{m}x_{\pi_{m}^{\left(l\right)}}+h_{\pi_{m}^{\left(l\right)}}^{†}v_{m}\left(\sum_{i=\pi_{m}^{\left(1\right)}}^{\pi_{m}^{\left(l-1\right)}}x_{i}\right)\\ \; & \;+h_{\pi_{m}^{\left(l\right)}}^{†}\left(\sum_{i\in [M]\backslash \{m\}}\sum_{j\in G_{i}}v_{i}x_{j}\right)+n_{\pi_{m}^{\left(l\right)}}. \end{array}$$

From (20), we obtain the user $\pi_{m}^{\left(l\right)}$’s SINR as follows:

(21) $$\begin{array}{cc} {\mathrm{SINR}}_{\pi_{m}^{\left(l\right)}}\; & =\frac{P_{\pi_{m}^{\left(l\right)}}{\left|h_{\pi_{m}^{\left(l\right)}}^{†}v_{m}\right|}^{2}}{|h_{\pi_{m}^{\left(l\right)}}^{†}v_{m}{|}^{2}\cdot \sum_{i=1}^{l-1}P_{\pi_{m}^{\left(i\right)}}+\sum_{i\in [M]\backslash \{m\}}\sum_{j=1}^{|G_{i}|}P_{\pi_{i}\left(j\right)}{\left|h_{\pi_{m}^{\left(l\right)}}^{†}v_{i}\right|}^{2}+1}\\ \; & =\frac{P_{\pi_{m}^{\left(l\right)}}\Gamma_{\pi_{m}^{\left(l\right)}}}{\Gamma_{\pi_{m}^{\left(l\right)}}\cdot \sum_{i=1}^{l-1}P_{\pi_{m}^{\left(i\right)}}+\sum_{i\in [M]\backslash \{m\}}\sum_{j=1}^{|G_{i}|}P_{\pi_{i}\left(j\right)}{\left|h_{\pi_{m}^{\left(l\right)}}^{†}v_{i}\right|}^{2}+1}, \end{array}$$

where $\Gamma_{\pi_{m}^{\left(l\right)}}={\left|h_{\pi_{m}^{\left(l\right)}}^{†}v_{m}\right|}^{2}$ using the notation (5).

Meanwhile, when l=1, Equation (21) simply becomes

(22) $$\begin{array}{c} {\mathrm{SINR}}_{\pi_{m}^{\left(l\right)}}=\frac{P_{\pi_{m}^{\left(l\right)}}\Gamma_{\pi_{m}^{\left(l\right)}}}{\sum_{i\in [M]\backslash \{m\}}\sum_{j=1}^{|G_{i}|}P_{\pi_{i}\left(j\right)}{\left|h_{\pi_{m}^{\left(l\right)}}^{†}v_{i}\right|}^{2}+1}. \end{array}$$

For notational simplicity, in the denominator of (21), we define the inter-beam interference denoted by $I_{\mathrm{inter}\text{-}\mathrm{beam}}$ as follows

(23) $$\begin{array}{cc} I_{\mathrm{inter}\text{-}\mathrm{beam}} & ≜\sum_{i\in [M]\backslash \{m\}}\sum_{j=1}^{|G_{i}|}P_{\pi_{i}\left(j\right)}{\left|h_{\pi_{m}^{\left(l\right)}}^{†}v_{i}\right|}^{2}. \end{array}$$

Then, for low-latency NOMA, our proposed random beam-based NOMA allocates the same power for each beam, i.e.,

(24) $$\begin{array}{c} \sum_{i=1}^{|G_{m}|}P_{\pi_{m}}^{\left(i\right)}=\frac{P}{M}\;\;\;\mathrm{for}\mathrm{all}\;\;\;m\in \left[M\right], \end{array}$$

so that the transmitter can omit the power allocation across the beams, which requires more feedback overhead and heavy computational complexity.

With the equal beam power allocation (24), the inter-beam interference at the user $\pi_{m}^{\left(l\right)}$ given in (23) is reduced to

(25) $$\begin{array}{cc} I_{\mathrm{inter}\text{-}\mathrm{beam}}\; & ≜\sum_{i\in [M]\backslash \{m\}}\sum_{j=1}^{|G_{i}|}P_{\pi_{i}\left(j\right)}{\left|h_{\pi_{m}^{\left(l\right)}}^{†}v_{i}\right|}^{2}\\ \; & =\frac{P}{M}\cdot \sum_{i\in [M]\backslash \{m\}}{\left|h_{\pi_{m}^{\left(l\right)}}^{†}v_{i}\right|}^{2}\\ \; & =\frac{P}{M}\cdot \left(∥h_{\pi_{m}^{\left(l\right)}}{∥}^{2}-\Gamma_{\pi_{m}^{\left(l\right)}}\right). \end{array}$$

Thus, the SINR given in (21) becomes

(26) $$\begin{array}{c} {\mathrm{SINR}}_{\pi_{m}^{\left(l\right)}}=\frac{P_{\pi_{m}^{\left(l\right)}}\Gamma_{\pi_{m}^{\left(l\right)}}}{\Gamma_{\pi_{m}^{\left(l\right)}}\cdot \sum_{i=1}^{l-1}P_{\pi_{m}^{\left(i\right)}}+\frac{P}{M}\cdot \left(∥h_{\pi_{m}^{\left(l\right)}}{∥}^{2}-\Gamma_{\pi_{m}^{\left(l\right)}}\right)+1}. \end{array}$$

Note that with given power allocation, the SINR of the user $\pi_{m}^{\left(l\right)}$ in (26) is only represented by both the user’s channel gain (i.e., ${∥h_{k}∥}^{2}$) and the effective channel gain with the selected beam (i.e., $\Gamma_{k}$). This fact means that each user’s feedback information should be the selected beam index, the corresponding effective channel gain, and the channel gain, i.e., for user *k*, the feedback information becomes

(27) $$\begin{array}{c} \{I\left(k\right),\;\Gamma_{k},\;∥h_{k}{∥}^{2}\}. \end{array}$$

Thus, given sub-user grouping $\left\{G_{1},\ldots ,G_{M}\right\}$ and the power allocation

(28) $$\begin{array}{c} \left\{P_{\pi_{m}}^{\left(1\right)},\ldots ,P_{\pi_{m}}^{\left(|G_{m}|\right)}\;|\;m\in \left[M\right]\right\}, \end{array}$$

the transmitter’s achievable sum rate with the target SINR γ becomes

(29) $$\begin{array}{c} R_{\mathrm{sum}}^{\mathrm{NOMA}}=\sum_{m=1}^{M}\sum_{l=1}^{|G_{m}|}\left[1\left({\mathrm{SINR}}_{\pi_{m}^{\left(l\right)}}\geq \gamma \right)\cdot \log_{2}\left(1+\gamma \right)\right]. \end{array}$$

## 4. Optimization of the Proposed Random Beam-Based NOMA

In this section, we optimize our proposed random beam-based NOMA.

To maximize the sum achievable rate in (29), we need to find the optimal sub-user groups and the optimal power allocation as follows

P1:maximizeG1,…,GM∈[K]{Pπm(1),…,Pπm(|Gm|)}m=1M∑m=1M∑l=1|Gm|1(SINRπm(l)≥γ)·log2(1+γ)subjecttoGi∩Gj=ϕwheneveri≠j,∑i=1|Gm|Pπm(i)=P/Mforallm∈[M].

Then, as we can observe in (26), the power allocation at each beam is independent of the allocated power for the other beams. Thus, the problem P1 can be divided into independent *M* sub-problems, each of which corresponds to user selection and power allocation for each beam. The transmitter only selects users and allocates powers for each beam; for the *m*th beam, the transmitter solves the following problem:

P2:maximizeGm∈[K],Pπm(1),…,Pπm(|Gm|)∑l=1|Gm|1(SINRπm(l)≥γ)·log2(1+γ)subjectto∑i=1|Gm|Pπm(i)=P/M.

Thus, the problem P1 can be solved by Algorithm 1. First, the transmitter initializes the sub-user groups $G_{1}^{\prime },\ldots ,G_{M}^{\prime }$ from the users’ selected beam indexes in the feedback information. For the *m*th beam, the initial sub-user group $G_{m}^{\prime }$ becomes

(30) $$\begin{array}{c} G_{m}^{\prime }=\left\{I(k)=m\;|\;k\in [K]\right\}. \end{array}$$

Then, the transmitter finds the initial decoding order $\pi_{m}^{\left(1\right)},\ldots ,\pi_{m}^{\left(|G_{m}^{\prime }|\right)}$ from the relationship (18). From (21), the transmitter finds the first user’s power allocation to satisfy the target SINR such that

(31) $$\begin{array}{c} {\mathrm{SINR}}_{\pi_{m}^{\left(1\right)}}=\gamma , \end{array}$$

which is given by

(32) $$\begin{array}{c} P_{\pi_{m}^{\left(1\right)}}=\frac{\gamma }{\Gamma_{\pi_{m}^{\left(1\right)}}}\cdot \left[\frac{P}{M}\left(∥h_{\pi_{m}^{\left(l\right)}}{∥}^{2}-\Gamma_{\pi_{m}^{\left(1\right)}}\right)+1\right]. \end{array}$$

Then, the transmitter can find $P_{\pi_{m}^{\left(2\right)}},\ldots ,P_{\pi_{m}^{\left(|G_{m}^{\prime }|\right)}}$ to satisfy the target SINR as follows:

(33) $$\begin{array}{c} P_{\pi_{m}^{\left(l\right)}}=\gamma \sum_{i=1}^{l-1}P_{\pi_{m}^{\left(i\right)}}+\frac{\gamma }{\Gamma_{\pi_{m}^{\left(l\right)}}}\cdot \left[\frac{P}{M}\left(∥h_{\pi_{m}^{\left(l\right)}}{∥}^{2}-\Gamma_{\pi_{m}^{\left(l\right)}}\right)+1\right],\;\;l=2,\cdots ,\left|G_{m}\right|. \end{array}$$

Then, the transmitter finds the largest $G_{m}\subset G_{m}^{\prime }$ to satisfy the power constraint as follows:

(34) $$\begin{array}{c} \sum_{j\in G_{m}}P_{j}\leq \frac{P}{M}. \end{array}$$

This procedure is described in Algorithm 1.

**Algorithm 1:** The proposed solution of our random beam-based NOMA.

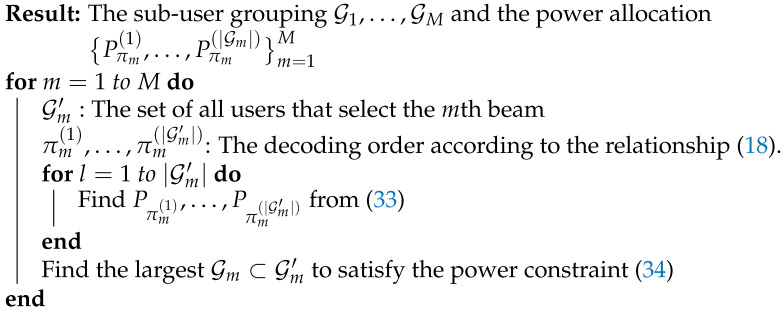



## 5. Numerical Result

In this section, we evaluate our proposed random beam-based NOMA.

**Figure 2:**
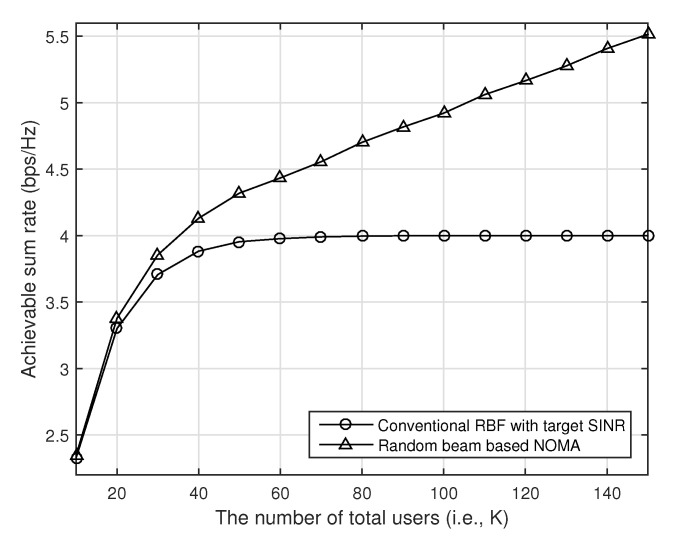
The performance of our proposed random beam-based NOMA with respect to the number of users when the transmitter has four antennas, and SNR is 10 dB.

In Figure 2, we compare the achievable sum rate of the conventional random beamforming and our proposed random beam-based NOMA with respect to the number of users when the number of transmit antennas is four, and the transmit SNR is 10 dB (i.e., P=10). The target SINR is fixed to one, i.e., γ=1. As we can see in Figure 2, the performance of the conventional random beamforming is saturated as the number of users increases because the target SINR is achieved for all beams. Since the transmitter can support at most four users, the maximum achievable rate with the conventional random beamforming becomes $M\log_{2}\left(1+\gamma \right)=4$. In our proposed random beam-based NOMA, however, the achievable rate increases as the number of users increases because our proposed scheme can support more users at each beam with NOMA.

**Figure 3:**
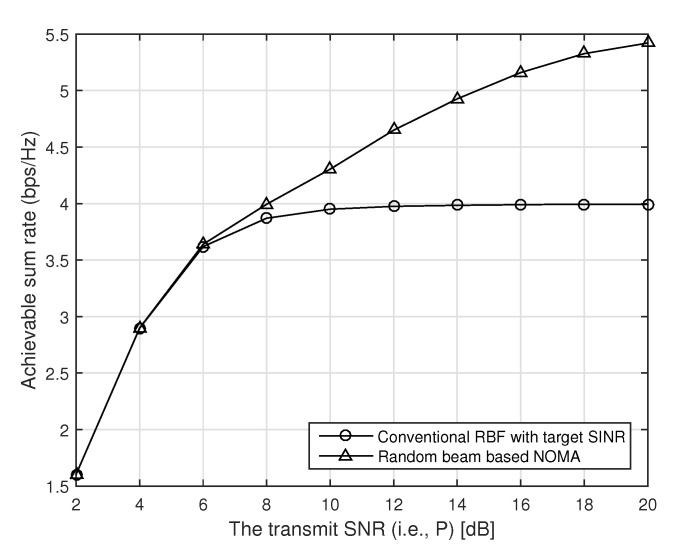
The performance of our proposed random beam-based NOMA with respect to the transmit SNR when the transmitter has four antennas, and the number of users is 50.

In Figure 3, we compare the achievable sum rate of the conventional random beamforming and our proposed random beam-based NOMA with respect to the transmit SNR when there are total fifty users (i.e., K=50), and the target SINR is fixed to one, i.e., γ=1. As we can see in Figure 3, the performance of the conventional random beamforming is saturated as target SINR is achieved as the transmit SNR increases. In our proposed random beam-based NOMA, however, the achievable rate increases as the transmit power increases because each beam can support more users with more transmit power.

**Figure 4:**
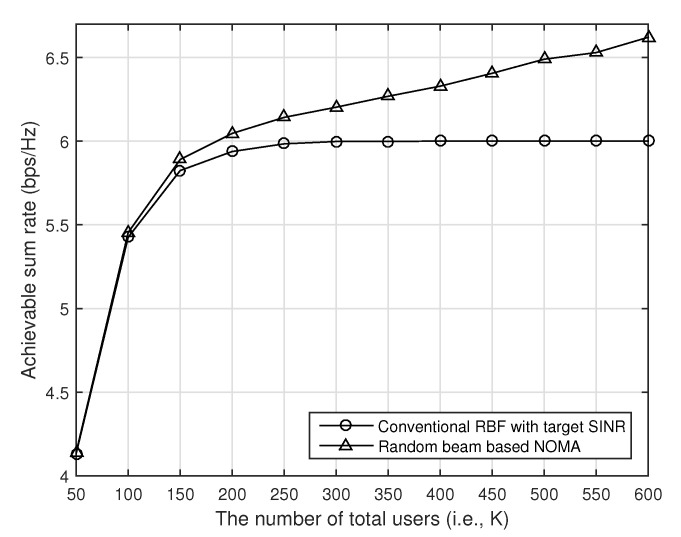
The performance of our proposed random beam-based NOMA with respect to the number of users when the transmitter has six antennas, and SNR is 10 dB.

In Figure 4, we compare the achievable sum rate of the conventional random beamforming and our proposed random beam-based NOMA with respect to the number of users when the number of transmit antennas is six, and the transmit SNR is 10 dB (i.e., P=10). The target SINR is fixed to one, i.e., γ=1. As we can see in Figure 4, the performance of the conventional random beamforming is saturated as the number of users increases because the target SINR is achieved for all beams. In this case, the transmitter can support at most six users, so the maximum achievable rate with the conventional random beamforming becomes $M\log_{2}\left(1+\gamma \right)=6$. In our proposed random beam-based NOMA, however, the achievable rate increases as the number of users increases because our proposed scheme can support more users at each beam with NOMA.

**Figure 5:**
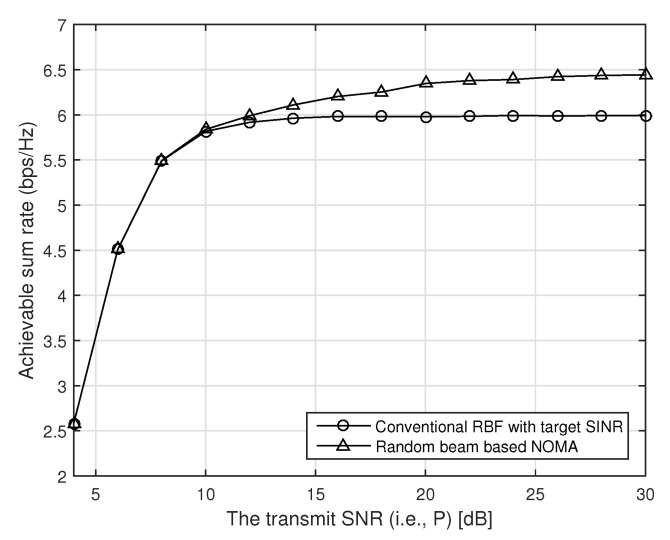
The performance of our proposed random beam-based NOMA with respect to the transmit SNR when the transmitter has six antennas, and the number of users is 200.

In Figure 5, we compare the achievable sum rate of the conventional random beamforming and our proposed random beam-based NOMA with respect to the transmit SNR when the number of transmit antennas is six (i.e., M=6), and there are total 200 users (i.e., K=200). In this case, the target SINR is fixed to one, i.e., γ=1. As we can see in Figure 5, the performance of the conventional random beamforming is saturated as target SINR is achieved as the transmit SNR increases. In our proposed random beam-based NOMA, however, the achievable rate increases as the transmit power increases because each beam can support more users with more transmit power.

**Figure 6:**
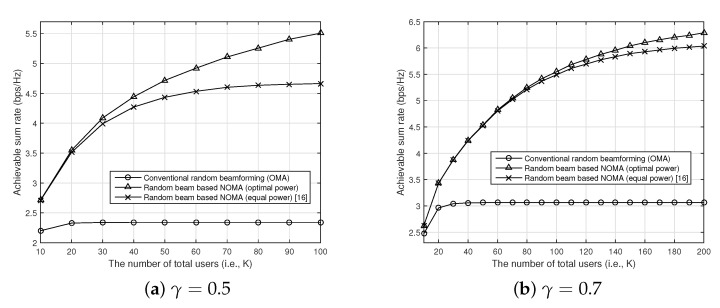
The achievable sum rate of our proposed random beam-based NOMA with respect to the number of users for various target SINRs (i.e., γ) when the transmitter has four antennas, and SNR is 10 dB.

In Figure 6, we show the achievable sum rate of our proposed random beam-based NOMA with respect to the number of users for various target SINR (i.e., γ) when the transmitter has four antennas, and SNR is 10 dB. As shown in Figure 6, our proposed random beam-based NOMA improves the conventional random beamforming when the target SINR is fixed by supporting multiple users for each beam. Furthermore, we can observe that the effect of the optimal power allocation becomes larger when the target SINR for each user is small. This is because with the smaller target SINR, each beam can support more users with NOMA.

## 6. Conclusions

In this paper, we proposed random beam-based NOMA for low latency MISO BCs, where there exists a target SINR for each user. For low latency, the transmitter exploits random beams, so each user can reduce the channel feedback overhead, while each beam can support more than a single user with NOMA. We established the joint optimization problem of user scheduling and power allocation for random beam-based NOMA. By allocating equal powers across the beams, we reduced the feedback overhead from each user, and showed that the joint optimization can be divided into several sub-optimization problems at all beams. We found the optimal power allocation and user scheduling for each sub-optimization problem, and we showed that our proposed random beam-based NOMA increases the performance of the conventional random beamforming by supporting more than a single user at each beam.

## Data Availability

Not applicable.
